# Tenth International Medical Congress, Berlin, 1890

**Published:** 1890-01

**Authors:** 


### Tenth International Medical Congress, Berlin, 1890.

RULES OF ORGANIZATION.

I. The sessions of the Tenth International Medical Congress will be opened in Berlin, Monday, August 4, 1890, and will close on August 10, 1890.

II. The membership of the congress is made up of legally qualified medical men, who are registered and have obtained their cards of admission. Other scientists who are interested in the work of the congress may be admitted to associate membership. The participants pay a registration fee of $5. They receive a copy of the transactions as soon as published. Registration should be made at the commencement of the meeting. Persons may also register previous to the meeting by sending the registration fee, their names, stating the position they hold and the place of residence.

III. The objects of the congress are for purely scientific purposes.

IV. The work of the congress is distributed in eighteen sections. At the time of registration, persons should designate the section or sections of which they wish to become members.

V. The committee on organization will conduct the election of permanent officers, which shall consist of a president, three vice-presidents, and of a number of honorary presidents and secretaries.

The various sections will elect their officers, consisting of a president and a number of honorary presidents. The latter are to conduct the sessions of the sections in conjunction with the president. For the reason that a number of different languages are official, it will be necessary to select a suitable number of secretaries from among the foreign delegates. Their duties are confined to the sessions of the congress.

After the close of the congress the transactions are to be published by an editorial committee especially appointed for the purpose.

VI. Sessions of the congress and of the separate sections will be announced later.

VII. The general sessions are: *a* For the transaction of business relating to the general affairs of the congress. *b*. For papers and communications of general interest.

VIII. Transactions of the sections will be announced later.

IX. Papers will be read at the regular general sessions as well as at special regular sessions, if any are held, by those only who have been selected for this purpose by the committee on organization. Suggestions relating to the prospective work of the congress should be in the hands of the committee on organization on or before July 1, 1890. The latter will decide whether the suggestions should be accepted or acted upon.

X. All essays, papers or communications read before the general sessions or the various sections, must be delivered in writing to the respective secretaries at the close of each session.

The committee on publication decides whether papers shall be published whole, or in part, or at all.

Members who take part in the discussions are requested to give the secretaries a written copy of their remarks before the close of each day.

XI. The official language of the congress is German, French and English. The rules, as well as the programs and daily announcements, will be published in all three languages. It is permissible for members to avail themselves of any other language, for the purpose of making brief remarks, provided some one is present to translate them into one of the official languages of the congress.

XII. Papers read before the sections are as a rule limited to twenty minutes; while speakers during discussion are limited to ten minutes each.

XIII. The presiding officer conducts the session according to the usual parliamentary rules governing the holding of similar meetings.

XIV. Students of medicine and others, ladies or gentlemen, who are not physicians, but who take special interest in the proceedings of the various sections, may be invited by the presiding officer, or be permitted, on application, to attend the sessions as visitors.

XV. Communications or requests for information relating to any one section should be addressed to the president thereof. Send other communications or apply for information to the general secretary, Dr. Lassar, Karlstrasse 19, Berlin, N. W., Germany.

The committee of organization of Section 14, Dental and Oral Surgery, consists of the following named gentlemen: Dr. Busch, Berlin, secretary; MM. Calais, of Hamburg; Hesse, of Leipsig; Fricke, of Kiel; Hollænder, of Halle; Miller, of Berlin; Pætsch of Breslau; Sauer, of Berlin, and Weil, of Munich.

